# Extracellular vesicles from activated Vδ2 T cells inhibit viral replication and enhance adaptive antiviral immunity

**DOI:** 10.1186/s12967-026-08062-9

**Published:** 2026-04-10

**Authors:** Veronica Bordoni, Federica Guarracino, Francesco Marocco, Luca Quattrocchi, Angela Lorusso, Antonella Minutolo, Sabrina Garbo, Marsha Pellegrino, Mara Vinci, Claudia Matteucci, Daria Pagliara, Federica Galaverna, Pietro Merli, Cecilia Battistelli, Franco Locatelli, Chiara Agrati

**Affiliations:** 1 Unit of Pathogen-Specific Immunity, Department of Hematology/Oncology, Cell and Gene Therapy, Bambino Gesù Children’s Hospital, IRCCS, Rome, Italy; 2 Department of Molecular Medicine, Sapienza University of Rome, Rome, Italy; 3 Department of Experimental Medicine, University of Rome Tor Vergata, Rome, Italy; 4 Research Unit of Genetics and Epigenetics of Paediatric Tumours, Bambino Gesu’ Children’s Hospital, IRCCS, Rome, Italy; 5 Department of Hematology/Oncology, Cell and Gene Therapy, Bambino Gesù Children’s Hospital IRCCS, Rome, Italy; 6 Saint Camillus International University of Health Sciences, Rome, Italy; 7 Catholic University of the Sacred Heart Rome, Rome, Italy; 8 Pathogen-Specific Immunity, Department of Hematology and Oncology, Bambino Gesù Children’s Hospital – IRCCS, Rome, Italy

**Keywords:** Vδ2 T cells, Extracellular vesicles, Antiviral response, Immunocompromised patients, MicroRNA

## Abstract

**Background:**

Vδ2 T cells are promising candidates for approaches of immunotherapy due to their unique pleiotropic functions; they were recently shown to enhance antiviral protection in hematopoietic stem cell transplantation (HSCT) recipients via innate effector activity and modulation of virus-specific adaptive T-cell response. Extracellular Vesicles (EVs) are key carriers of immunomodulatory signals and Vδ2-derived EVs (Vδ2-EVs) exhibit antitumor activity but their role in viral infection remain unclear. The aim of this study was to investigate the direct and immunomodulatory antiviral functions of Vδ2-EVs in healthy subjects and HSCT patients.

**Methods:**

The direct antiviral activity of Vδ2-EVs were tested in vitro using a model of Cytomegalovirus (CMV) replication. The immunomodulatory antiviral activities of Vδ2-EVs were evaluated in both healthy donors and HSCT recipients by functional immunological assays (cytokine release and proliferation capability of virus-specific T cells). Finally, their molecular cargo was characterized through miRNA sequencing.

**Results:**

Our findings reveal that Vδ2-EVs efficiently inhibit CMV replication, reducing the frequency of CMV-infected fibroblast cells. Moreover, Vδ2-EVs are taken up by myeloid cells and were able to activate antigen-presenting cells, leading to an increased frequency of CMV-specific T cells, as measured by IFN-γ production. Accordingly, Vδ2-EVs enhanced the proliferation of CMV-specific T-cell clones in HSCT pediatric recipients. Finally, the analysis of miRNA content in Vδ2-EVs highlighted the enrichment of miRNAs that target genes regulating critical antiviral response processes such as SOCS1.

**Conclusions:**

Altogether, this study provides new insights into the antiviral functions of Vδ2-EVs and underscores their translational therapeutic potential as modulators of antiviral immunity in immunocompromised settings.

**Supplementary Information:**

The online version contains supplementary material available at 10.1186/s12967-026-08062-9.

## Introduction

γδ T cells are key players in immunity against cancer and infectious diseases, acting as a bridge between the innate and adaptive immune systems [1–3]. These cells are divided into two major subsets based on Vδ chain usage: the predominant Vδ2 subset and the non-Vδ2 subsets [4]. In contrast to conventional αβ T cells, Vδ2 T cells recognize low-molecular-weight phosphoantigens (PhAgs) in an MHC-independent manner via a mechanism involving CD277/butyrophilin-3A1 (BTN3A1) [5]. This unique function enables them to serve as an early warning system against pathogens and transformed cells. Upon activation, Vδ2 cells display pleiotropic effector functions, including potent cytotoxicity against a wide range of tumor cells and broad antiviral activity mediated through both cytolytic and non-cytolytic mechanisms [4, 6]. They can be easily activated in vivo by aminobisphosphonates (e.g. zoledronic acid) or by a recently optimized monoclonal antibody ICT01 targeting BTN3A1 (NCT04243499) [7, 8]. For these reasons, Vδ2 T cells are considered promising targets for immunotherapeutic strategies, and several oncology clinical trials testing their use are currently underway (www.clinicalstrial.gov*).*

Optimizing antitumor and antiviral immune responses represents a critical goal following hematopoietic stem cell transplantation (HSCT). During the early post-transplant period, patients experience profound immunodeficiency, which increases their susceptibility to infections, viral reactivations, and cancer relapse, thereby contributing to higher morbidity and mortality [9]. We recently proposed a possible contribution of Vδ2 T cells in mediating antiviral protection in pediatric recipients of haploidentical HSCT (haplo-HSCT), and demonstrated their interplay with adaptive virus-specific αβ T-cell immunity [10]. In these patients, we identified a Vδ2 T-cell signature associated with protection from viral reactivation, characterized by their ability to directly inhibit viral replication and to provide adjuvant support to virus-specific αβ T-cell responses. Moreover, we demonstrated that these functional properties are at least partially mediated by soluble factors released by PhAg-stimulated Vδ2 T cells, consistent with a bystander mechanism driven by Vδ2-derived soluble mediators.

Extracellular Vesicles (EVs) represent key mediators in non–contact-dependent cell-to-cell communication: cells release EVs as regulated molecular cargo (DNA, RNA, proteins, lipids) carriers, thus playing a central role in cell-cell crosstalk [11]. In recent years, they have attracted growing scientific interest due to their potential wide clinical applications [12]. Specifically, with respect to the role of EVs in immunomodulation, available evidence indicates that EVs produced by Vδ2 T-cells directly kill cancer cells and deliver immunomodulatory signals to enhance the anticancer adaptive immunity [13]. Indeed, it has been reported that γδ T cell-derived EVs may act as a promising immunotherapeutic platform for the treatment of cancers, particularly those associated with Epstein-Barr virus and nasopharyngeal carcinoma [13, 14]. Despite the well-recognized antiviral properties of Vδ2 T cells, the contribution of Vδ2 derived EVs to antiviral immunity remains largely unexplored.

The aim of this study was to characterize the antiviral and immunomodulatory properties of Vδ2-derived EVs (Vδ2-EVs) in healthy donors (HD) and haplo-HSCT. Moreover, we defined EVs miRNAs molecular content conferring the observed immunomodulatory properties, thus relating the EVs structural features with their functional abilities.

Our data indicate that Vδ2 T cell–derived EVs inhibit CMV replication and improve the cytokine production and proliferation capabilities of αβ virus-specific T cells both in HD and in haplo-HSCT recipients. Moreover, next-generation sequencing of Vδ2-derived EV-miRNA cargo identified seven enriched miRNAs able to specifically modulate key genes involved in the antiviral response.

## Methods

### Patients and donors

To study the immunomodulatory properties of Vδ2-EVs, healthy donors (HD, *n* = 10) and pediatric recipients of Haplo-HSCT (*n* = 10) were enrolled at Bambino Gesù Children’s Hospital in a cross-sectional study. Specifically, pediatric patients (median age 8.5 years, IQR 5–9.7; 4 females and 6 males) who underwent TCRαβ/CD19-depleted haplo-HSCT for malignant disease [B-cell Acute lymphoblastic leukemia (*n* = 7), T-cell Acute lymphoblastic leukemia (*n* = 7), refractory cytopenia off childhood (*n* = 2)] and who were fully engrafted, alive, and disease-free at day 180 were enrolled in this study. The TCRαβ/CD19-depleted haplo-HSCT is characterized by the infusion not only of a high number of CD34 + hematopoietic stem and progenitor cells, but also of a high number of γ/δ T cells and natural killer (NK) cells. The project was approved by the Ethics Committee of the Bambino Gesù Children’s Hospital, Rome, Italy (Approval number 3135/2023).

### Generation of Vδ2-T cell lines

Peripheral blood mononuclear cells (PBMCs), isolated from Buffycoats were stimulated with HDMAPP (1 µg/mL, Echelon Biosciences Inc, Salt Lake City, USA) in RPMI-1640 medium supplemented with 10% FBS (Euroclone, Milan, Italy), 2 mM glutamine (Euroclone) and 1% penicillin/streptomycin (Euroclone). The cultures were maintained at 37 °C, 5% CO₂ for two weeks supplemented with IL-2 (2 µg/mL, Bio-Techne, Minneapolis, USA), and IL-15 (0.04 µg/mL, Bio-Techne). The purity of the Vδ2 T cell subset was assessed by flow-cytometry and found to be consistently higher than 90%. To proceed with the production and purification of EVs, the medium was removed and replaced with RPMI medium supplemented with 10% EV-depleted serum (Gibco, Grand Island, New York, USA). Cells were therefore re-stimulated with HDMAPP for 48 h and culture supernatants were collected to isolate the EVs derived from Vδ2 T cells (Vδ2-EVs). As a control, EVs were also generated from unstimulated PBMCs (ctr-EVs).

### EVs isolation

EVs have been purified(as in [15]) in accordance with the Minimal Information for Studies of Extracellular Vesicles (MISEV2023) guidelines [16]. Vδ2 T-cell–conditioned medium was centrifuged at 4 °C, 20 min 2,000 g to remove debris and aggregates, then at 4 °C, 30 min at 20,000 g to eliminate micro-vesicles, filtered (0.2 μm), and finally ultracentrifuged at 4 °C for 70 min at 100,000 g using the Optima MAX-XP Ultracentrifuge (Beckman Coulter, Brea, CA, USA) in an SW-32 Ti rotor (Beckman Coulter) to isolate EVs. Finally, EVs were re-suspended in PBS and washed at 4 °C for 70 min, at 100,000 g using the Optima MAX-XP Ultracentrifuge (Beckman Coulter) in an SW-32 Ti rotor (Beckman Coulter). The protein concentration of EVs was determined by Bradford assay using Bio-Rad protein assay dye Reagent concentrate (Bio-Rad Laboratories, Hercules, CA, USA). The size distribution of EVs was determined by EXOID-V1-SC (Izon Science Ltd., Christchurch, New Zealand) analysis based on tunable resistive pulse sensing (TRPS). Vδ2-EVs flow-cytometry and Western blot characterization and labeling are described in supplementary section.

### MRC-5 infection and characterization

Human fetal lung fibroblasts MRC-5 (from ATCC, Manassas, USA) were pre-treated with Vδ2-EVs or Ctr-EVs for 48 h and then infected with the HCMV strain AD169 in EMEM (ATCC) at a multiplicity of infection (MOI) of 1. Viral inoculum or medium only (non-infected control) was added, and cells were incubated for 1 h at 37 °C with 5% CO₂. After removing of inoculum, cells were washed and subsequently cultured at 37 °C with 5% CO₂ for 72 h. At the end of culture, cells were washed and processed for flow-cytometry analysis or for relative quantification of miRNA target genes’ expression. For flow-cytometry analysis, cells were stained for IE⁺ cells using a transcription factor/intranuclear staining kit (FOXP3/Transcription Factor Buffer Set, Thermo Fisher Scientific, Massachusetts, USA), according to the manufacturer’s instructions. Briefly, cells were fixed for 30 min at 4 °C, washed twice and stained with an anti-HCMV IE-1 antibody (Thermo Fisher Scientific, 1:500) for 40 min at 4 °C. After washing, cells were incubated with an Alexa Fluor 555-conjugated secondary antibody (Thermo Fisher Scientific; 1:500 in Permeabilization Buffer) for 40 min at 4 °C, washed again, and acquired on a BD FACSLyric (BD Biosciences, San José, California, USA). FACS data were analyzed using FlowJo Software (Version 10).

For gene expression analysis, MRC-5 cells at 72 h post-infection were lysed in Buffer RLT (RNeasy micro kit, Qiagen, Hilden, Germany) supplemented with 1% beta-mercaptoethanol (Sigma-Aldrich, Darmstadt, Germany); RNA was extracted accordingly with the manufacturer’s instructions and analyzed as reported in the RT-qPCR analysis section.

### Vδ2-EVs uptake in MRC5 cells lines and in immune cell subsets

MRC-5 cell lines and PBMCs from HD were labelled with CellTrace™ Far Red Cell Proliferation Kit (Thermo Fisher Scientific) following the manufacturer’s protocol. Vδ2-EVs were labeled with PKH67 (Sigma-Aldrich) as described in the Supplementary section. PKH67-labeled Vδ2-EV were then incubated overnight with Far-Red-stained MRC5 and PBMC at 37 °C, 5% CO₂, into an appropriate imaging 96 well black plate. After 18 h [14], MRC5 and PBMC (stained with anti-CD14 BV421, BD Biosciences), were analyzed on the Operetta CLS (PerkinElmer, Shelton, Connecticut, USA). A flow-cytometry analysis was also performed on PBMC to quantify the specific homing in the different immune subsets. Briefly, PKH67-labeled Vδ2-EV were incubated overnight with PBMC; then PBMC were stained with CD3- BV480,CD19- APC (both from BD Biosciences), and CD14-V450 (Miltenyi Biotec, Bergisch Gladbach, Nord Reno-Westfalia, Germany), and analyzed by flow-cytometry on a BD FACSLyric.

### ELISpot assay for IFN-γ detection

ELISpot assays were performed using the Human IFN-γ ELISpot Plus (HRP) kit (Mabtech, Cincinnati, USA) following the manufacturer’s instructions. Briefly, PBMCs from HD were pretreated with or without Vδ2-EVs (20 µg/mL) for 24 h. Thereafter, cells were stimulated for 24 h with CMV pp65 peptide pools (JPT Peptide Technologies, Berlin, Germany) at 0.1, 0.01, and 0.001 µg/mL at 37 °C, 5% CO_2_. Spot-forming cells (SFCs) were counted with an automated ELISpot reader (IRIS Elispot Reader), and results were expressed as SFCs per 10⁶ PBMCs after subtraction of background from unstimulated control wells. As a negative control, PBMCs from HIV-negative HD were stimulated with HIV peptide pools (JPT Peptide Technologies) under the same conditions.

### Functional immune assays

To assess monocyte and dendritic cell (DC) activation, DCs (obtained as described in the supplementary section) or total PBMCs were incubated for 24 h at 37 °C and 5% CO₂, with or without Vδ2-EVs (20 µg/mL), and then analyzed by flow-cytometry after staining with CD11c-PE-Cy7 (Immunological Sciences, Texas, USA), HLA-DR- APC-H7 (BD Biosciences), and CD14-V450 (Miltenyi Biotec).

Moreover, Vδ2-EVs-pretreated DCs were co-cultured with autologous PBMCs at a 1:10 DCs: PBMCs ratio and stimulated with CMV pp65 peptide pools (1 µg/mL, JPT Peptide Technologies) for 7 days at 37 °C, 5% CO₂. T-cell proliferation was quantified by measuring the frequency of Ki-67 + T cells by flow cytometry. Cells were first surface-stained with CD3- Pe-Cy7 (BD Biosciences) for 15 min at 4 °C in BD Stain Buffer, then fixed with BD Cytofix for 20 min at 4 °C, permeabilized with BD Perm/Wash and stained with Ki-67 -Alexa Fluor 700 (BD Biosciences) for 30 min at 4 °C. Samples were acquired on a BD FACSLyric and analyzed with FlowJo Software (Version 10).

The proliferation assay was also performed using total PBMC from haplo-HSCT pediatric recipients. Briefly, Vδ2-EVs-pretreated PBMC were stimulated with CMV pp65 peptide pools (1 µg/mL, JPT Peptide Technologies) for 7 days and the proliferation of T cells was measured as described above.

### Small RNA sequencing and data analysis

miRNAs samples were sequenced at Procomcure Biotech GmbH. Sequencing libraries were prepared using the NEXTFLEX Small RNA-Seq Kit v4 (PerkinElmer, Waltham, MA, USA). The sequencing reaction was performed on an Illumina NovaSeq 6000 instrument in 2 × 40 bp paired-end configuration, with a throughput of ~ 40 million read pairs per sample. FastqToolkit (version 2.2.5) was used to remove adapter sequences from the 3’ end, whereas Cutadapt (version 4.5) was used to filter out reads whose length and average quality after trimming were < 16 and < 30, respectively. Only forward reads were kept for downstream analyses. Bowtie (version 1.0.0) and a custom Python script developed in-house were used to respectively align and quantify the reads against a reference composed of mature miRNA sequences downloaded from miRBase v22.1 database. The mapped counts were normalized using the trimmed mean of M values (TMM) normalization method to account for differences in library size and composition between samples. Differential expression of miRNAs between conditions was then assessed by applying the quasi-likelihood F-test (QLF) implemented in edgeR. Genes with a false discovery rate (FDR) < 0.05 were considered significantly differentially expressed.

### Target prediction & functional enrichment analyses

The prediction of the genes targeted by the microRNAs and the subsequent functional analysis were performed using R (version 4.3.1) pipeline developed in-house. In detail, the prediction of miRNA target genes was performed using the multiMiR package (version 1.24.0), to obtain a high-confidence target list, containing only miRNA-mRNA interactions predicted by at least two independent databases among the ones included in multiMiR.

Gene Ontology (GO) and Kyoto Encyclopedia of Genes and Genomes (KEGG) enrichment analyses were performed using clusterProfiler package, to identify biological processes (BP) and pathways significantly overrepresented among the target genes. For both analyses, p-values were adjusted for multiple testing using the Benjamini–Hochberg false discovery rate (FDR) correction. Terms or pathways with an adjusted p-value < 0.05 were considered statistically significant. To focus on processes relevant in the viral and immune response, enriched GO terms and KEGG pathways were filtered to retain only those functionally associated with viral and immune response.

### Quantitative real-time PCR for the validation of microRNA sequencing

miRNAs were extracted by miRNeasy Mini Kit and RNeasy MinElute Cleanup Kit (Qiagen, Hilden, Germany) (as in [17]) accordingly to the manufacturer’s protocol, and reverse transcribed with microScript microRNA cDNA Synthesis Kit (Norgen Biotek Corp., Thorold, ON, Canada). Quantitative polymerase chain reaction (RT-qPCR) analyses were performed according to MIQE guidelines. cDNAs were amplified by qPCR reaction using GoTaq qPCR Master Mix (A600A; Promega, Madison, WI, USA). miRNA relative amounts, obtained with the 2^(-ΔCt) method, were normalized with respect to the cel-miR-39 (Spike-In Norgen Biotek Corp., Thorold, ON, Canada), previously added into miRNA samples proportionally to the miRNA concentration. The primers sequences for miRNA validation are reported in supplementary materials and methods.

### RT-qPCR analysis

Total RNA was extracted from cells using RNeasy micro kit (Qiagen, Hilden, Germany) and cDNA was synthesized using the iScriptTM c-DNA Synthesis Kit (Bio-Rad Laboratories Inc., Hercules, CA, USA) according to the manufacturer’s instructions. RT-qPCR was performed (as in [18]) using GoTaq qPCR Master Mix (A600A; Promega, Madison, WI, USA). Relative amounts, obtained with 2(-ΔCt) method, were normalized with respect to the housekeeping gene L32. The primer sequences are reported in supplementary materials and methods.

### Statistical analyses

Two-tailed Mann–Whitney U-test, and Wilcoxon matched-pairs signed rank test were used to compare continuous variables as appropriate (indicated in Figures). A p value < 0.05 was considered statistically significant. Statistical analyses were all performed using GraphPad Prism 10 (La Jolla, CA, USA).

## Results

### Vδ2-EVs characterization

**Fig. 1:**
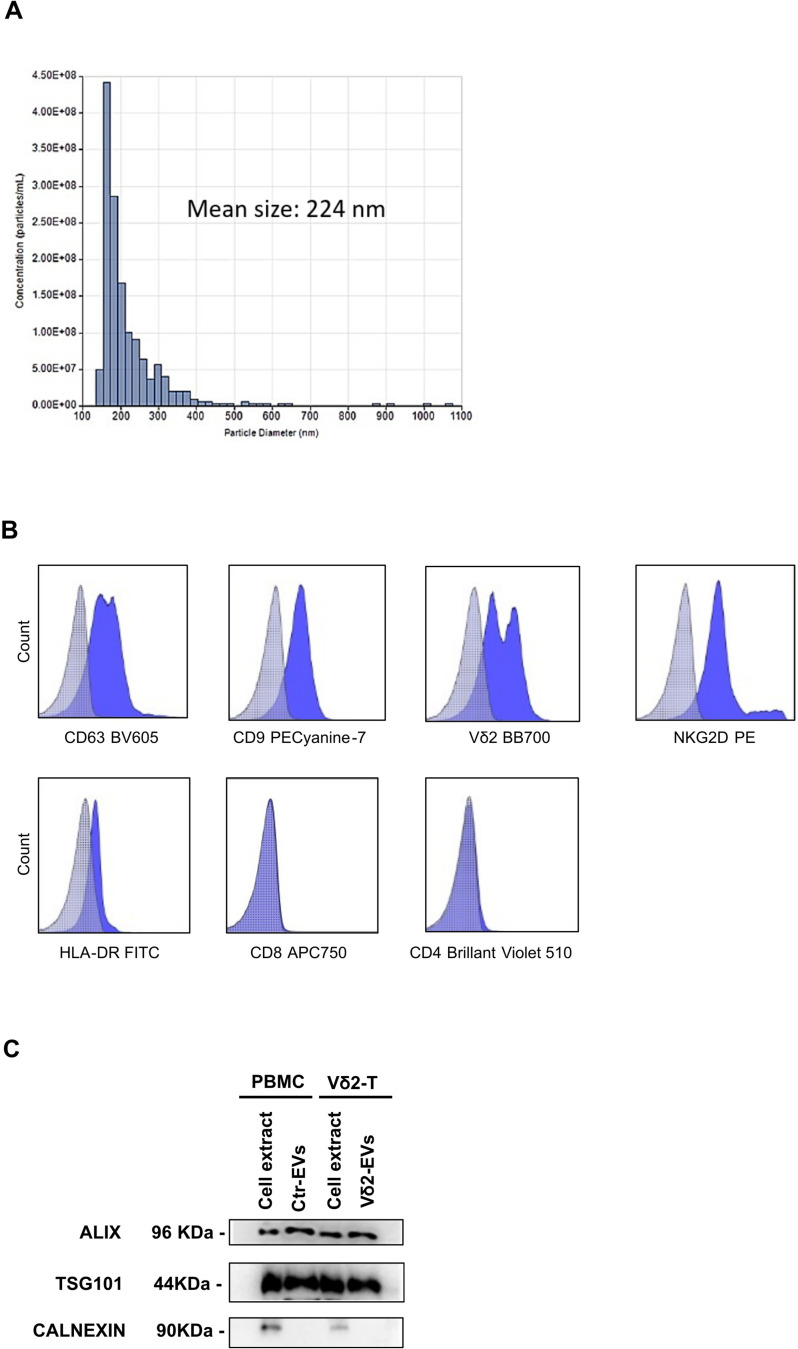
Characterization of Vδ2-EVs. (**A**) Representative graph showing the size distribution of human Vδ2-EVs measured by EXOID-V1-SC. (**B**) Intensity of surface staining for the indicated functional molecules on Vδ2-EVs was determined by flow-cytometry. Light-blue dotted histograms represent isotype controls. Representative data are shown (*n* = 5). (**C**) Exosomal markers TSG101 and Alix, along with the endoplasmic reticulum marker calnexin, were detected in PBMCs and Vδ2-T cells and in their respective EVs (ctr-EVs and Vδ2-EVs) by Western blot analysis

Vδ2-EVs were isolated by ultracentrifugation and quantified. Tunable resistive pulse sensing (TRPS) analysis showed a bell-shaped size distribution profile with a peak at ~ 200 nm (Fig. 1A). Flow-cytometry analysis showed that Vδ2-EVs express CD63, CD9, and molecules typically found on activated Vδ2 T cells, such as Vδ2-TCR and NKG2D, HLA-DR, while lacking CD4 and CD8 expression (Fig. 1B). The expression of positive (Alix and TSG101) and negative (Calnexin) EVs markers was confirmed by Western Blot analysis (Fig. 1C**)**.

### Vδ2-EVs display direct antiviral activity on CMV-infected MRC5 cells

**Fig. 2:**
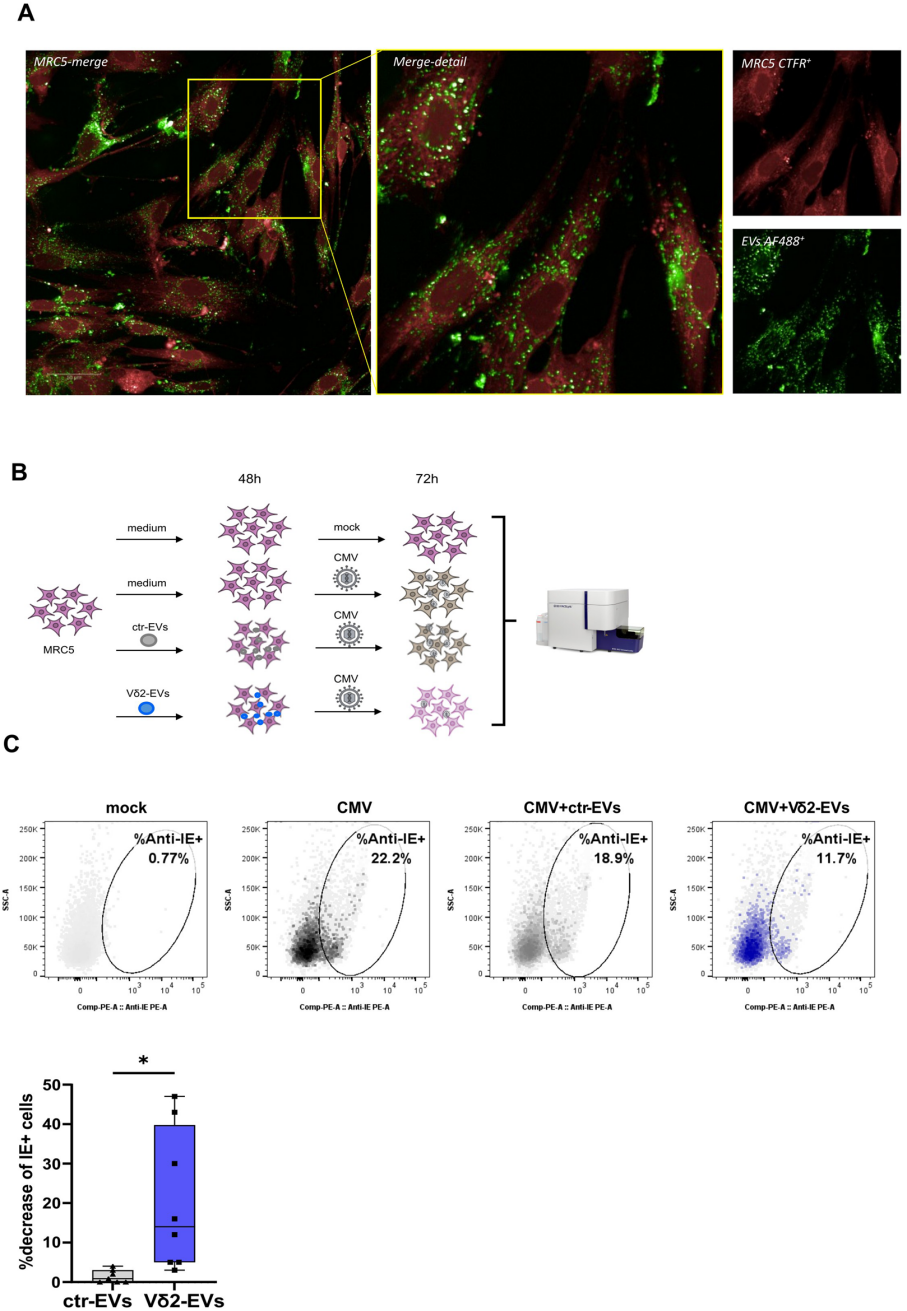
Vδ2-EVs inhibit CMV replication. (**A**) Confocal microscopy (scale bar = 50 μm) showing Cell-Trace Far Red-stained MRC5 (red) incubated with PKH67-Labeled Vδ2-EVs (green). (**B**) Experimental design. MRC5 were pretreated with ctr-EVs (grey), or with Vδ2-EVs (blue) for 48 h or maintained in the culture medium without stimulation (medium). Then, MRC5 were infected with CMV and the frequency of infected cells were quantified after72 h by flow-cytometry. (**C**) Representative flow-cytometry dot plots showing the frequency of IE-1 positive MRC5 are shown. Results are expressed as median and interquartile range (*n* = 8). Statistical analysis was performed using a paired t test: *p < 0.05

Previous findings showed that factors secreted by activated Vδ2 T cells can inhibit CMV replication [10], suggesting a potential role of molecules packaged within Vδ2-derived EVs in mediating this antiviral effect. To test this hypothesis, we verified the ability of PKH67-labeled Vδ2-EVs to be up-taken by MRC5 cells by confocal microscopy (Fig. 2A), and we measured their effect on CMV replication. Specifically, MRC5 cells were pretreated for 48 h with Vδ2-EVs or ctr-EVs and then infected with AD169 CMV (MOI 1). After 72 h from infection the frequency of CMV-infected MRC5 was measured by monitoring the percentage of IE+ MRC5 cells by flow-cytometry (Fig. 2B). Uninfected MRC5 cells (mock) were used as negative control. We demonstrated that the treatment with EVs did not induce MRC5 cell death (data not shown). Interestingly, Vδ2-EVs induced a reduction in the percentage of infected cells (IE-1 + MRC5) compared with untreated CMV-infected MRC5 cells; this reduction was significantly higher than that observed with ctrl-EVs (Fig. 2C).

### Vδ2-EVs are efficiently taken up by monocytes and induce their activation

**Fig. 3:**
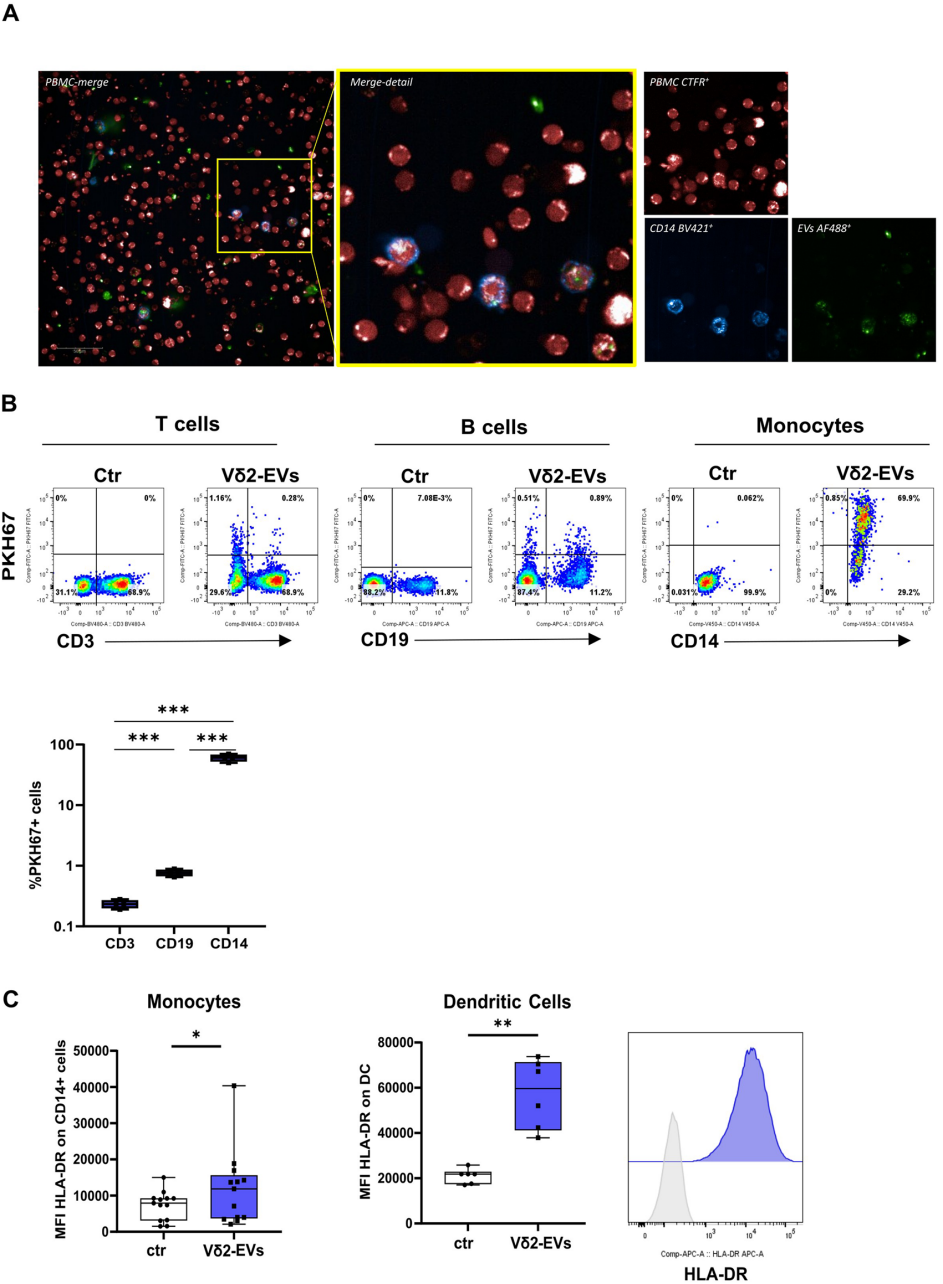
Cellular tropism of Vδ2-EVs. (**A**) Confocal microscopy (scale bar = 50 μm) showing Cell-Trace Far Red-stained PBMC (red) incubated with PKH67-Labeled Vδ2-EVs (green) and stained with anti-CD14 monoclonal antibody (blue). (**B**) Representative flow-cytometry dot plots+ showing the uptake of Vδ2-EVs (PKH67, green) in T (CD3+), B (CD19+) and monocytes (CD14+) cells. Results are expressed as median and interquartile range (*n* = 5). Statistical analysis was performed using paired test: ***p < 0.001. (**C**) The expression of HLA-DR on monocytes and DC was quantified in resting and Vδ2-EVs stimulated cells. Results are expressed as median and interquartile range of HLA-DR expressed as the mean of fluorescence intensity (MFI) (*n* = 6). Representative histogram is shown. Statistical analysis was performed using paired Wilcoxon and paired t test: *p < 0.05, **p < 0.01

Before assessing the functional immunomodulatory properties of Vδ2-EVs, their specific tropism toward circulating immune cell subsets was explored. PKH67-labeled Vδ2-EVs were incubated with freshly isolated PBMCs for 18 h. As a control (ctrl), PBMCs were cultured under the same conditions in the absence of Vδ2-EVs. Confocal microscopy showed a preferential tropism of Vδ2-EVs for myeloid CD14⁺ cells (Fig. 3A), and flow-cytometry analysis confirmed that 60.5% (IQRs: 52.5–67.7) of CD14⁺ cells internalized Vδ2-EVs, whereas the uptake by B or T cells was less than 1% (Fig. 3B). To assess the biological effects on Vδ2-EVs on myeloid cells, whole PBMC, as well as monocyte-derived-DCs, were treated with Vδ2-EVs for 24 h. Results showed that Vδ2-EVs promoted an increased expression of the MHC-II surface receptor HLA-DR in both myeloid cell types, suggesting a possible contribution in the modulation of their antigen presenting capability (Fig. 3C).

### Vδ2-EVs improve the virus-specific T cell response in both HD and HSCT patients

**Fig. 4:**
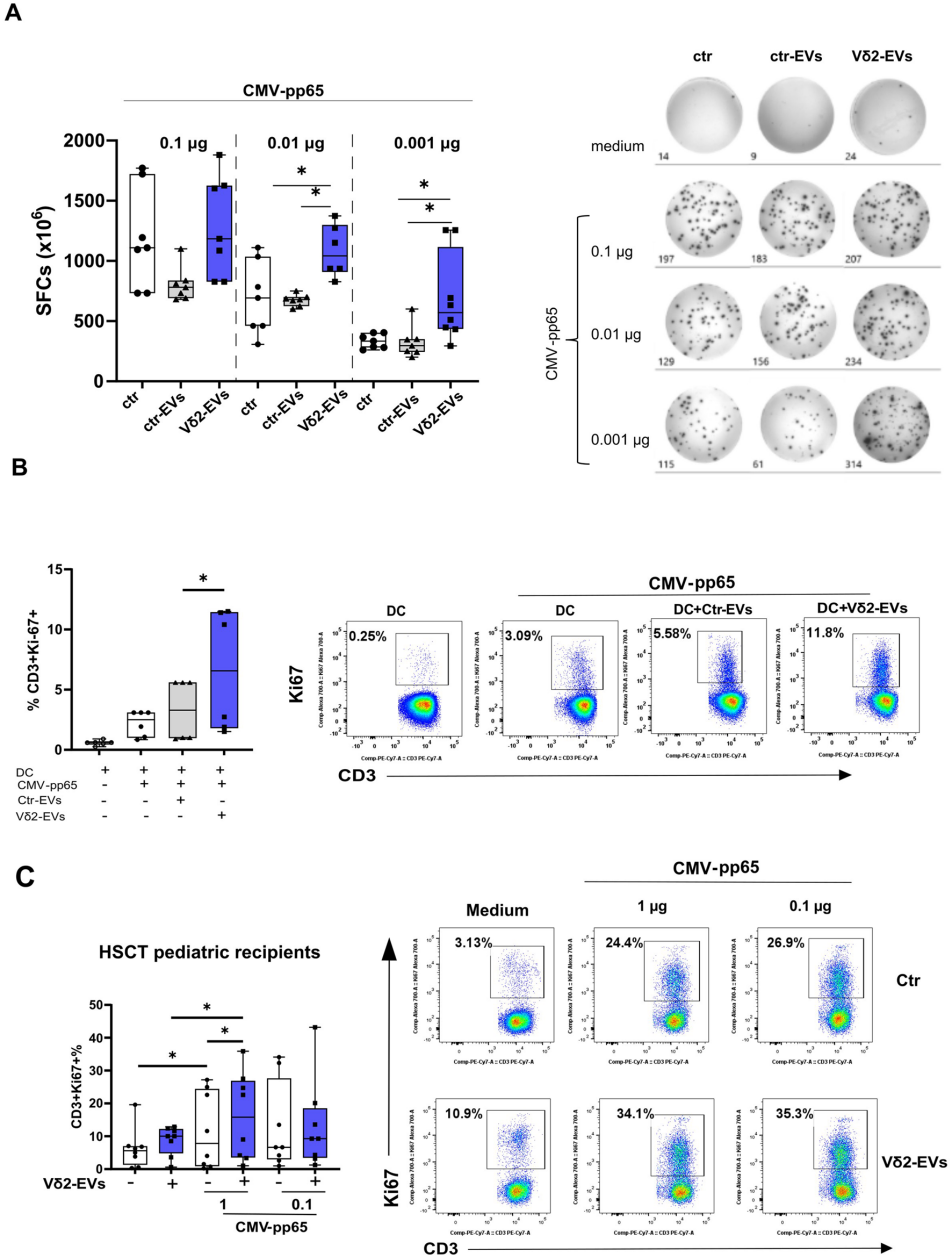
Vδ2-EVs improve viral-specific T-cell response. (**A**) The frequency of IFN-γ producing cells were evaluated by Elispot assay. PBMC were primed with Vδ2-EVs (blue boxes) or ctr-EVs (grey boxes) and then stimulated with decreasing concentration of CMV peptides (0.1, 0.01 and 0.001 µg/ml). Results are shown as median and interquartile range (*n* = 8). Representative ELISpot images are shown. Statistical analysis was performed using paired t test *p < 0.05. (**B**) The frequency of proliferating T cells from HD was assessed in the presence of autologous DCs pulsed with either Vδ2-EVs (blue boxes) or ctr-EVs (grey boxes) after 7 days of CMV stimulation (1 µg/ml). T cells alone and T cells with DC without CMV stimulation (white boxes) are used as controls. Results are shown as median and interquartile range (*n* = 6). Representative flow-cytometry dot plots are shown. Statistical analysis was performed using paired Wilcoxon test *p < 0.05. (**C**) The frequency of proliferating T cells was assessed in PBMCs from pediatric haplo-HSCT recipients, either unpulsed (white boxes) or pulsed with Vδ2-EVs (blue boxes), after 7 days of stimulation with CMV peptides. Results are shown as median and interquartile range (*n* = 10). Representative flow-cytometry dot plots are shown. Statistical analysis was performed using paired t test *p < 0.05

To assess whether Vδ2-EVs can modulate the virus-specific T-cell response, PBMCs isolated from CMV-seropositive HD were cultured with allogeneic Vδ2-EVs or ctrl-EVs for 24 h and then stimulated with different concentration of CMV peptides (0.1, 0.01 and 0.01 ug/ml) for 18 h. Unrelated peptides (HIV peptides) were used as negative control (data not shown). As shown in Fig. 4A, the frequency of IFN-γ producing T cells decreases proportionally to the decrease in CMV peptide concentration (blue boxes). Interestingly, under suboptimal peptide concentrations, Vδ2-EVs significantly increased the frequency of IFN-γ–producing T cells (Fig. 4A), supporting a role for Vδ2-EVs in enhancing the activation of CMV-specific T cells under suboptimal viral stimulation conditions.

Given the preferential tropism of Vδ2-EVs for monocytes, we next investigated whether the enhancement of the antigen-specific response was mediated through their effects on antigen presenting cells (APCs). To address this hypothesis, DCs were pretreated with Vδ2-EVs for 24 h and then co-cultured with autologous CD3⁺ T cells for one week in the presence of CMV peptides (1 µg/ml). The results showed that Vδ2-EVs-pulsed DCs were able to better present CMV antigen to T cells, resulting in a higher frequency of proliferating (Ki-67+) T cells (Fig. 4B). These data support the hypothesis that Vδ2-EVs improves the capacity of DCs to stimulate autologous virus-specific T cell proliferation.

The ability of EVs to enhance the antiviral response opens up several applications and may be of particular interest in the context of immunocompromised patients. We therefore evaluated the immunomodulatory properties of Vδ2-EVs in pediatric haplo-HSCT recipients using a proliferation assay on total PBMCs to better reflect physiological conditions. Priming of PBMCs from pediatric haplo-HSCT recipients with Vδ2-EVs enhanced the expansion capacity of CMV-specific T cells, suggesting their potential to support the proliferation of virus-specific T cells even in immunocompromised patients (Fig. 4C).

### Vδ2- EVs contain multiple antiviral and immune-regulating miRNAs

**Fig. 5:**
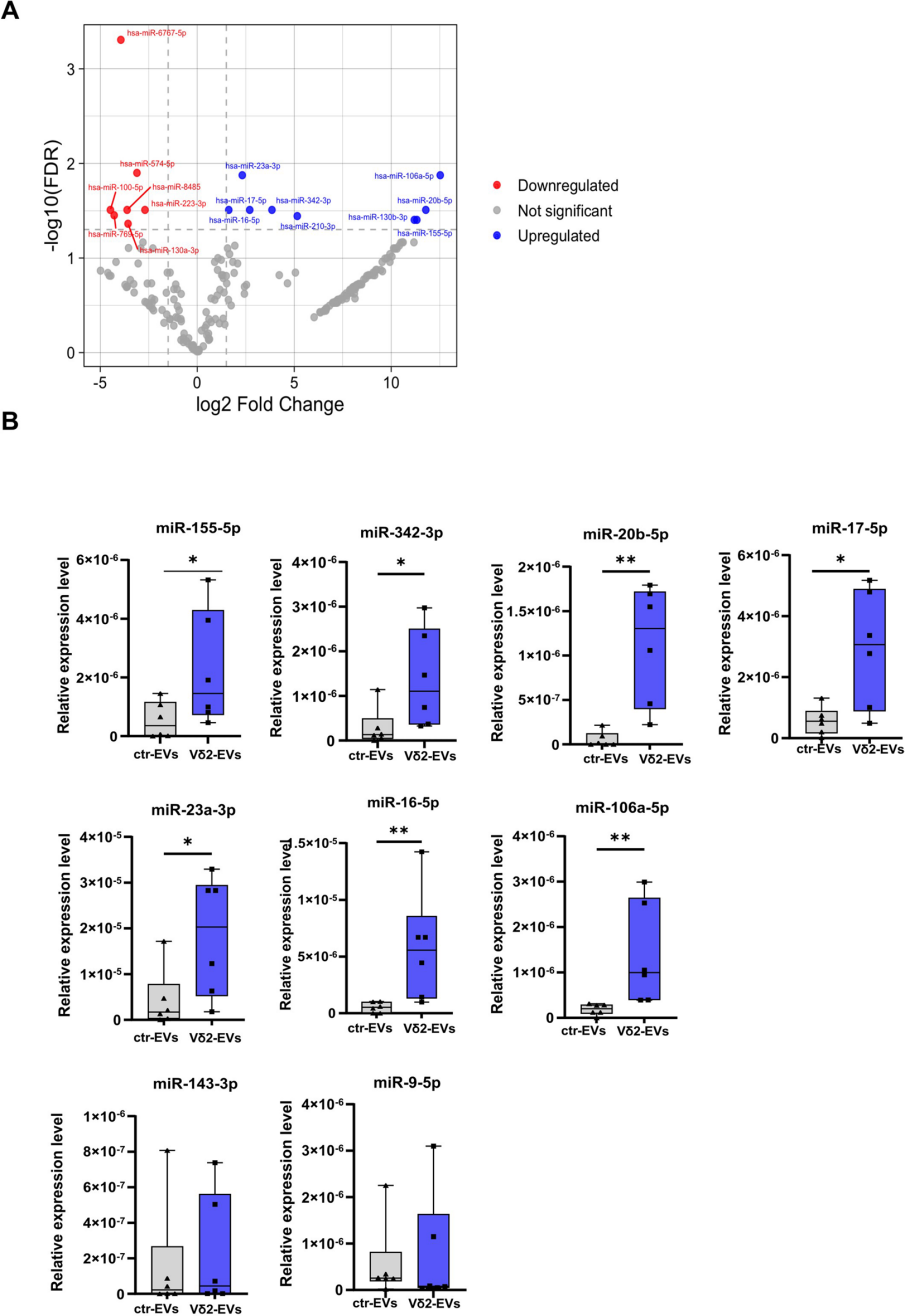
Vδ2-EVs contain immunomodulatory and antiviral miRNAs. (**A**) Volcano plot (right panel) highlights differential miRNA expression between Vδ2-EVs and ctrl-EVs (reference). Specifically, upregulated miRNAs are shown in red, while downregulated miRNAs are shown in blue. (**B**) Seven upregulated miRNAs and two not differentially enriched miRNAs were validated by RT-PCR in miRNA samples purified from Vδ2-EVs (blue boxes) and ctr-EVs (grey bars). Results are shown as median and interquartile range (*n* = 6). Statistical analysis was performed using Mann-Whitney test: * *p* < 0.05, ** *p* < 0.01

To investigate how the molecular cargo of Vδ2-EVs mediate their functional activity, we performed miRNA sequencing. Differential miRNA profiling between Vδ2-EVs and ctr-EVs identified a defined subset of miRNAs that is selectively enriched in Vδ2-EVs, as illustrated by the volcano plot (Fig. 5A). Notably, among the most abundant miRNAs carried by Vδ2-EVs, many converge on shared regulatory pathways (Table 1) that shape the amplitude, quality, and persistence of innate and adaptive immune responses (e.g., miR-106a-5p, miR-20b-5p, miR-155-5p, miR-23a-3p), and comprise miRNAs with documented direct antiviral activity (e.g., miR-342-3p, miR-17-5p-5p, miR-16-5p). Conversely, other miRNAs highly expressed in ctr-EVs, (e.g. miR-6767-5p and miR-574-5p), were found under-represented in Vδ2-EVs (Fig. 5A). RT-qPCR validation of the NGS data confirmed the enrichment of these miRNAs in Vδ2-EVs (Fig. 5B), indicating that Vδ2-EVs carry a selective miRNA cargo with potential roles in immunoregulatory and antiviral responses. As a control, we also tested two miRNAs (miR-143-3p and miR-9-5p) that were not differentially expressed in the volcano plot analysis.

**Table 1:** Functional description of significantly upregulated miRNAs in Vδ2-EVs categorized by their antiviral nature

	miRNA	Antiviral activity	FDR	Ref
Immune regulatory	hsa-miR-106a-5p hsa-miR-20b-5p	- Influencing antigen presentation - Modulating immune cell function	0,013	[28–29]
	hsa-miR-155-5p	- Targeting negative regulators of IFN - Modulating antiviral signaling	0,039	[30–32]
	hsa-miR-23a-3p	- Controlling Th differentiation	0,013	[34–35]
Critical for viral replication	hsa-miR-342-3p	- Inhibiting viral replication	0,031	[36]
	hsa-miR-17-5p	- Direct targeting of viral genome	0,031	[37–39]
	hsa-miR-16-5p	- Direct targeting of viral replication - Regulating cell apoptosis	0,031	[41]

FDR: False Discovery Rate; Ref: references

### Functional analysis of Vδ2-EVs -associated miRNA targets

**Fig. 6:**
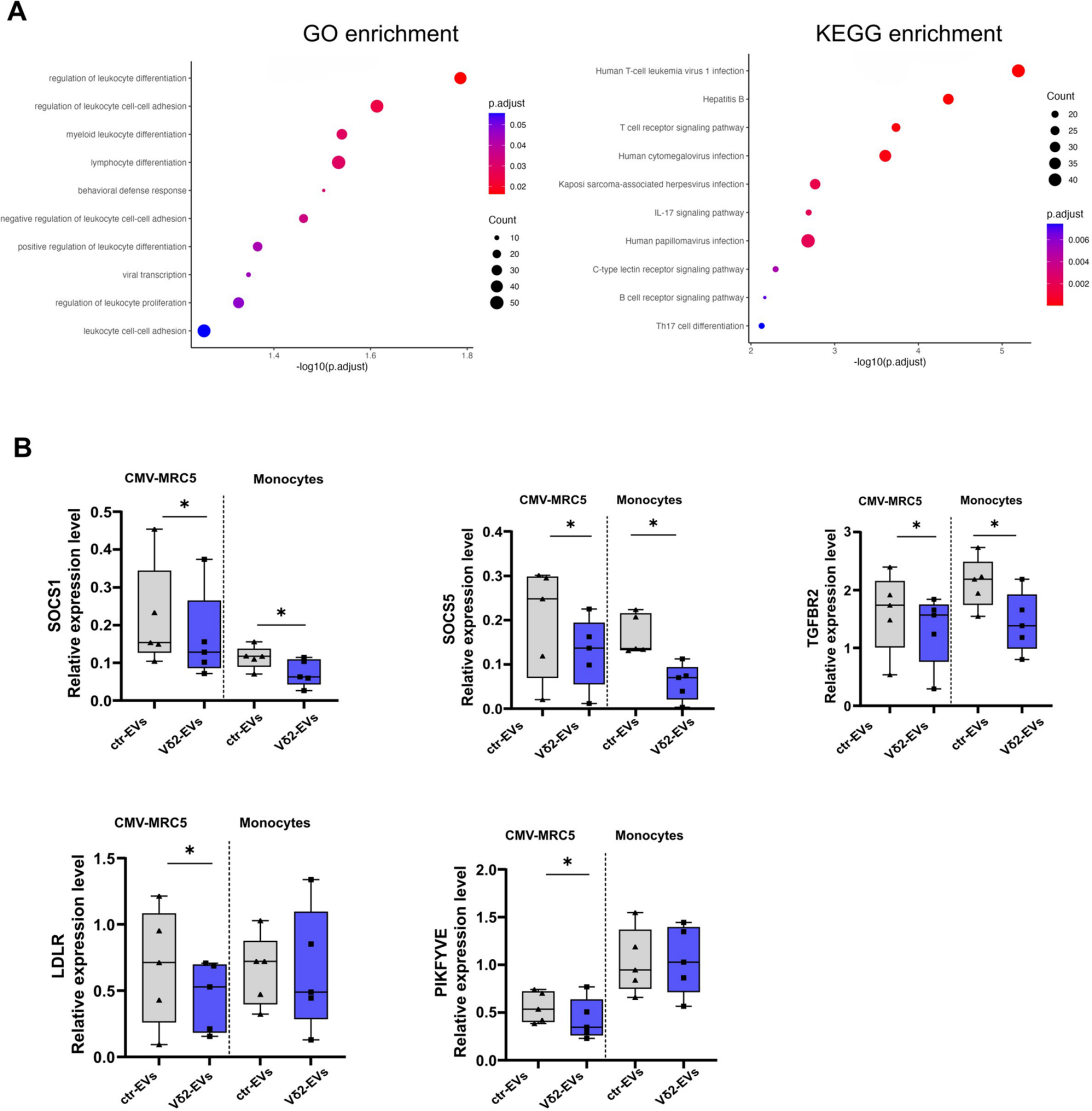
miRNA pathway enrichment and validation of miRNAs target genes. (**A**) GO term pathway enrichment (left panel) and KEGG pathway enrichment (right panel) for differentially expressed miRNAs in Vδ2-EVs. (**B**) The relative expression levels of miRNA target genes were quantified in CMV-infected MRC5 cells and monocytes pulsed with Vδ2-EVs (blue boxes) or ctr-EVs (grey bars). Results are shown as median and interquartile range (*n* = 5). Statistical analysis was performed using a paired test: *P* < 0.05

The exploration of the different pathways impacted by the identified Vδ2-EV-loaded miRNAs was performed by GO and KEGG analyses on the predicted targets of these miRNAs (Fig. 6A). GO enrichment revealed strong over-representation of pathways central to the immune response, including leukocyte differentiation, leukocyte cell–cell adhesion, and regulation of immune cell proliferation. These terms highlight genes involved in shaping immune cell development, communication and activation. Additional enrichment of key immune signaling pathways (TGF-β, JAK–STAT, PI3K, NF-κB) further indicates that the gene set broadly influences the molecular programs governing immune activation and responsiveness. KEGG pathway analysis revealed strong enrichment of pathways associated with viral infections and immune response. Several viral infection pathways, including HTLV-1, HBV, CMV and HPV, are among the most significantly enriched categories, reflecting the involvement of genes broadly engaged in antiviral signaling, immune activation, and host–pathogen interactions. In parallel, multiple immune system pathways were highly enriched, including the T-cell receptor, IL-17 signaling pathways as well as C-type lectin receptor signaling and Th17 cell differentiation, underscoring a coordinated activation of innate and adaptive immune modules.

Collectively, these results highlight that Vδ2-EVs selectively carry specific miRNAs, whose predicted targets are enriched in pathways associated with immune modulation and antiviral responses.

### **Vδ2-EVs miRNA-mediated regulation of target genes**

To validate the bioinformatic predictions, quantitative analysis of miRNA target genes was performed in EV-targeted cells, including CMV-infected MRC5 cell lines and monocytes (Fig. 6B). We selected five predicted target genes of miRNA specifically enriched in Vδ2-EVs (miR-155-5p, miR-106a-5p, miR-17-5p, and miR-23a-3p) based on their roles in viral replication (LDLR and PIKFYVE) and immune responses (SOCS1, SOCS5, and TGFBR2). Specifically, we quantified these target genes in CMV-infected MRC5 cells or monocytes treated with Vδ2-EVs or ctr-EVs using qRT-PCR. After Vδ2-EVs stimulation, the expression of the target genes with immunomodulating properties (SOCS1, SOCS5 and TGFBR2) was significantly reduced in both MRC5 and monocytes (Fig. 6B). Furthermore, Vδ2-EVs treatment induced a reduction of the expression of target genes involved in viral replication (LDLR and PIKFYVE) only in CMV infected MRC5 cells, and not in monocytes, confirming the specificity of their activity. These results indicate that EVs released by Vδ2 T cells promote antiviral immunity by downregulating key genes that inhibit interferon-mediated responses, facilitate viral replication, and restrain antigen-specific T-cell proliferation.

## Discussion

In recent years, human γδ T cells have emerged as key protective effectors, contributing to both antitumor and antiviral immunity thanks to their strong cytotoxic and immunomodulatory functions [19]. The unique biological properties of γδ T cells, including their ability to recognize a broad range of antigens in an MHC-independent manner, their expression of NK-associated receptors, and their strong capacity for rapid expansion, make them highly attractive targets for immune-therapeutic strategies [20]. Two therapeutic strategies have been explored: (i) direct in vivo activation of Vδ2 T cells using aminobisphosphonates (e.g., zoledronic acid [21]) or the monoclonal antibody ICT01 [7], and (ii) infusion of ex vivo–expanded Vδ2 T cells in autologous or allogeneic settings [22]. However, clinical outcomes in clinical trials have so far been limited. The major barrier appears to be the in vivo microenvironment, which suppresses their effector functions and may impair their homing to tumor or to infected tissues.

In order to overcome these limitations, increasing evidence supports the use of ex vivo–produced EVs from HDs as an effector delivery tool. EVs offer several advantages: they constitute a cell-free platform with simpler and more cost-effective regulatory requirements than cellular therapies, are derived from healthy cells, and can more readily cross biological barriers, thus reaching target sites to exert their therapeutic effect [23]. Moreover, EVs may offer further significant therapeutic advantages due to their natural ability to transfer bioactive molecules, such as proteins, lipids, and nucleic acids to target cells, enabling them to improve the immune response. Notably, EVs derived from Vδ2 T cells (Vδ2-EVs) have been identified as a promising immunotherapeutic tool in cancer settings due to their ability to efficiently reach and kill cancer cells [14], but no data are available about their role in modulating the antiviral immune response.

Our study aims to investigate the potential of using Vδ2-EVs to enhance the antiviral response. In particular, we focused on the potential direct and bystander antiviral activities of Vδ2-EVs in HD and in pediatric haploHSCT recipients, as well as on elucidating the underlying molecular mechanisms. Here, our data show that Vδ2-EVs express functional molecules (e.g. NKG2D and class-II), can be efficiently taken-up by epithelial cells and exhibit a preferential tropism toward myeloid immune cells.

During viral infections, EVs have emerged as critical mediators of intercellular communication [24]. Here we present for the first time data relating to the ability of Vδ2-EVs to inhibit viral replication using a CMV infection model. Pre-treatment of CMV-infected epithelial cell lines with Vδ2-EVs reduced the frequency of cells harboring replicating CMV, suggesting their ability to carry molecular signals able to interfere with critical steps of viral replication. The potential antiviral activity of Vδ2-EVs, combined with their efficient in vivo biodistribution, represents significant advantages for targeting tissues involved in viral replication.

Alongside their direct antiviral activity, in this study, we demonstrate the ability of Vδ2-EVs to enhance the antiviral immune response. Pre-treatment of PBMCs with Vδ2-EVs resulted in a significant increase in the frequency of CMV-specific IFN-γ–producing T cells. Moreover, Vδ2-EVs activated DCs and consistently improved their ability to trigger CMV-specific proliferation of autologous T cells. These findings suggest that Vδ2-EVs may enhance antigen presentation efficiency, thereby lower the activation threshold of CMV-specific T cells and potentiate antiviral responses. Whether Vδ2-EVs directly transfer co-stimulatory molecules to the surface of DCs or modulate gene transcription by delivering their informational cargo requires further evaluation. Previous studies have demonstrated that Vδ2-EVs inherited the antitumor properties from their parental cells [14]. Vδ2-EVs targeted and efficiently killed tumor cells through FasL and TRAIL pathways, and promoted tumor-specific CD4 and CD8 T-cell expansion both in hematologic [14] and solid tumors [13]. Our findings further support the hypothesis that Vδ2-EVs can transfer immunostimulatory signals to recipient APCs and amplify antigen-specific T-cell responses. Here, we demonstrate for the first time the immunomodulatory potential of Vδ2-EVs in pediatric patients undergoing haplo-HSCT for hematologic malignancies, a clinical setting characterized by profound post-transplant immunosuppression and a high risk of infections and viral reactivations [25]. Harnessing Vδ2-EVs, with their combined antiviral and antitumor activity, offers a promising therapeutic approach for prophylaxis, treatment, or as an adjuvant to vaccination to boost immunogenicity. Moreover, Vδ2-EVs could also be exploited as a vaccine delivery platform, leveraging their full biological activity as previously proposed in oncology settings [26].

The structural characterization of the miRNA content carried by Vδ2-EV, showed the enrichment of several miRNAs with an important role in mediating the antiviral activity and promoting antigen presenting function. We classified them into two groups (Table 1) based on the features of their target genes. The first group (miR-106a-5p, miR-20b-5p, miR-155-5p, and miR-23a-3p) comprises immune-responsive miRNAs that fine-tune antiviral innate immunity by enhancing type I interferon signaling and promoting DC maturation. miR-106a-5p and miR-20b-5p sustain antiviral responses by repressing negative regulators of interferon pathways [27, 28]. Specifically, a direct immunomodulatory effect of these miRNAs has been demonstrated: transfection of DCs with miR-106a-5p and miR-20b-5p enhances their maturation and increases IFN-γ production by T cells [27]. miR-155-5p, broadly induced by viral infection or TLR activation, reinforces JAK/STAT signaling via SOCS inhibition [29–31] and supports T-cell proliferation by targeting arginase-2, a key suppressor of antiviral and antitumor responses [32]. In particular, a direct effect of miR-155-5p has been demonstrated, as miR-155-5p–transfected monocytes are able to promote IFN-γ production by T cells [33]. Finally, miR-23a-3p regulates genes critical for T-cell differentiation [34, 35]. The second group (miR-342-3p, miR-17-5p, and miR-16-5p) comprises miRNAs that regulate host pathways critical in modulating the viral replication processes. miR-342-3p modulates the sterol biosynthesis pathway [36], providing lipids essential for viral envelope formation, particle assembly, and release. miR-17-5p exerts antiviral effects against viruses including enterovirus, HIV-1, adenovirus, and CMV by targeting viral genomes or host factors necessary for replication [37–39], and it also reduces the suppressive potential of myeloid-derived suppressor cells, enhancing antiviral and antitumor immunity [40]. Specifically, knockdown of miR-17-5p using a specific siRNA has been shown to enhance viral replication, whereas transfection with the miR-17-5p significantly inhibits viral replication, highlighting a direct antiviral effect [40]. Finally, miR-16-5p restricts viruses such as Dengue and Enterovirus 71 by regulating apoptosis and innate immune pathways via targets like BCL2 and CCND1 [41]. Using gain-of-function approaches, the transfection of miR-16-5p mimics is able to directly inhibit viral replication [41].

We validated predicted and known target genes of the upregulated miRNAs enriched in Vδ2-EVs, focusing on those involved in antiviral mechanisms in infected cells and immunomodulatory functions in antigen-presenting cells. Kang et al. showed that the infection by Zaire Ebolavirus and SARS-CoV-2 can be prevented by inhibiting the expression of PIKFYVE [42], a predicted target of several miRNAs enriched in Vδ2-EVs (miR-155, miR-106, and miR-17). Notably, PIKFYVE was significantly reduced in CMV-infected MRC5 cells after treatment with Vδ2-EVs, highlighting a direct role of Vδ2-EVs in affecting the cell processes involved in viral replication. Accordingly, the analysis of LDLR expression, a critical molecule for viral entry [43] showed a significant downregulation of their expression in CMV-infected MRC5 cells pretreated with Vδ2-EVs.

We also observed downregulation of SOCS1 and SOCS5 (targets of miR-155 and miR-106) in both infected MRC5 cells and monocytes pretreated with Vδ2-EVs. This reduction is consistent with a stronger interferon-driven antiviral response expected to limit viral replication and to enhance antigen presentation [44, 45]. In line with this, Toniolo et al. reported that SOCS5 down modulation in antigen-presenting cells enhances antigen presentation, cytokine secretion, and T-cell priming, ultimately promoting a more effective antiviral adaptive immune response [46]. Finally, TGFBR2 (target of miR-106, miR-20-5p and miR-17-5p) was downregulated in monocytes pretreated with Vδ2-EVs. Loss of TGF-β signaling has been shown to enhance anti‐tumorigenic properties [47] and antiviral response [48], confirming the hypothesis of the immunoregulatory role of cargo of Vδ2-EVs.

## Conclusions

Overall, our results demonstrate that Vδ2-EVs, which carry multiple miRNAs with antiviral and immunomodulatory functions, can inhibit viral replication and enhance antiviral immune responses in both healthy and immunocompromised hosts, providing a proof of concept for their potential clinical application. Furthermore, the ability to load Vδ2-EVs with molecules of interest, such as viral antigens, cytotoxic agents, or inhibitors of suppressive pathways, offers a valuable strategy to further optimize immune responses in vulnerable patients.

## Supplementary Information

Below is the link to the electronic supplementary material.

Supplementary Material 1

## Data Availability

The data supporting this article’s findings are available from the corresponding author upon reasonable request.

## Abbreviations

HSCT: Hematopoietic stem cell transplantation
haplo-HSCT: Haploidentical HSCT
EVs: Extracellular Vesicles
Cytomegalovirus: CMV
PhAgs: Phosphoantigens
BTN3A1: CD277/butyrophilin-3A1
HD: Healthy donors
NK: Natural killer
PBMC: Peripheral blood mononuclear cells
TRPS: Tunable resistive pulse sensing
MO: Multiplicity of infection
GO: Gene Ontology (GO)
KEGG: Kyoto Encyclopedia of Genes and Genomes
FDR: False discovery rate
DC: Dendritic cells
HTLV-1: Human T-lymphotropic Virus type 1
HBV: Hepatitis B Virus
HPV: Human Papillomavirus
