# Supplementary material for: Extracellular vesicles from activated Vδ2 T cells inhibit viral replication and enhance adaptive antiviral immunity
Source: J Transl Med. 2026 Apr 10;24:524. doi: 10.1186/s12967-026-08062-9 (PMC13088707; doi:10.1186/s12967-026-08062-9)
Supplement: Supplementary file 1 — Supplementary Material 1 [file 12967_2026_8062_MOESM1_ESM.docx]

**Extracellular Vesicles from Activated Vδ2 T Cells Inhibit Viral Replication and Enhance Adaptive Antiviral Immunity**

Veronica Bordoni et al.

**Supplementary material and methods**

1. **Vδ2-EVs characterization**

Flow cytometer (CytoFLEX, Beckman Coulter) was used to identify the extracellular vesicles (EV) population according to size, as suggested by the guidelines on minimal information for the study of extracellular vesicles (MISEV 2024). For the analysis of EVs, a gating strategy was generated by using a combination of FITC-labelled fluorescent Megamix-Plus SSC (Biocytex, France) and Megamix-Plus FSC beads (Biocytex, France), hereby termed as Gigamix. The Gigamix contains beads of sizes 0.1 μm, 0.16 μm, 0.2 μm, 0.24 μm, 0.3 μm, 0.5 μm and 0.9 μm. Acquisition settings for EVs were adjusted as FSC gain at 170, SSC gain 100, vSSC gain at 500. Before the acquisition of EVs, the flow cytometer was cleaned with a cleaning buffer, and the filtered PBS was read before the reading of the sample. For phenotypic characterization, the EVs were incubated for 20 min at room temperature in the dark with 1:50 of Anti Human HLA-DR FITC (BioLegend, San Diego, USA), CD9 PECyanine-7 (SONY, Japan), CD63 BV605 (SONY), CD8 APC750 (BioLegend), CD4 Brillant Violet 510 (BioLegend), Vd2 BB700 (BD Biosciences, San José, California, USA), NKG2d PE (Miltenyi Biotec, Bergisch Gladbach, Nord Reno-Westfalia, Germany). The stained EVs were then washed with filtered 0,22 μm filter PBS, centrifuged at 13.000 RPM x 60 sec and resuspended in 50 μl filtered PBS for acquisition. The concentration and quantification of EVs (number of events/µl, total number of events and percentage) were calculated using CytoFLEX (Beckman Coulter) and CytExpert 2.3 software (Beckman Coulter), and 150000 events were acquired in the 0.1 and 0.9 μm range.

1. **Protein extraction and western-blot analysis**

Cells and EVs were lysed in Triton 1X Buffer, subsequently the proteins were analyzed as in (15). The following primary antibodies were used for immunoblotting: Calnexin Antibody (Novus Biologicals), Alix Antibody (Cell Signaling Technology), TSG101 Antibody (Santa Cruz Biotechnologies).The immune complexes were detected with horseradish peroxidase-conjugated species-specific secondary antiserum: (α-Rabbit and α-Mouse Bio-Rad Laboratories), then by enhanced chemiluminescence reaction (Bio-Rad Laboratories).

1. **Vδ2-EVs labeling**

Vδ2-EVs labeling was performed using the PKH67 Green Fluorescent Cell Linker Kit (Sigma-Aldrich, Hessen, Germany) according to the manufacturer’s instructions. Briefly, 1 µL of PKH67 dye was diluted in 1 mL of Diluent C (Sigma-Aldrich) to prepare the staining solution. Vδ2-EVs (20 µg/mL) were resuspended in the PKH67/Diluent C mix and incubated for 5 min at RT with gentle mixing. Unincorporated dye was removed by ultracentrifugation at 100000 g for 70 min, and Vδ2-EV pellet was resuspended in PBS and kept protected from light.

1. **Dendritic cells (DC) culture**

To generate immature monocyte-derived dendritic cells, monocytes were isolated from PBMCs of healthy donors by positive selection using anti-CD14 magnetic microbeads (Miltenyi Biotec), according to the manufacturer’s instructions as described in supplementary section. The purity of CD14⁺ cells was assessed by flow cytometry and averaged 96%. Monocytes were plated at 2 × 10⁵ cells/cm² in RPMI 1640 medium supplemented with 10% FBS (Euroclone, Milan, Italy), 2 mM glutamine, and 1% penicillin/streptomycin (all from Euroclone). Cultures were supplemented with GM-CSF and IL-4 (50 ng/mL and 10 ng/mL, respectively, both from Miltenyi Biotec). After one week, the dendritic cell phenotype was verified by flow cytometry using staining with CD11c-PE-Cy7 (Immunological Sciences, Texas, USA) CD1c-BB515, HLA-DR-APC-H7 (both from BD Biosciences), CD14-V450 (Miltenyi Biotec), and a lineage exclusion mix (CD3/CD19/CD56-APC, BD Biosciences).

1. **Quantitative real-time PCR for the validation of microRNA sequencing**

miRNAs were extracted by miRNeasy Mini Kit and RNeasy MinElute Cleanup Kit (Qiagen, Hilden, Germany) (as in 38878288, 40601477) accordingly to the manufacturer’s protocol, and reverse transcribed with microScript microRNA cDNA Synthesis Kit (Norgen Biotek Corp., Thorold, ON, Canada). Quantitative polymerase chain reaction (RT-qPCR) analyses were performed according to MIQE guidelines. cDNAs were amplified by qPCR reaction using GoTaq qPCR Master Mix (A600A; Promega, Madison, WI, USA). miRNA relative amounts, obtained with the 2^(-ΔCt) method, were normalized with respect to the cel-miR-39 (Spike-In Norgen Biotek Corp., Thorold, ON, Canada), previously added into miRNA samples proportionally to the miRNA concentration. The primers sequences for miRNA validation are reported in primer sequences section.

1. **RT-qPCR analysis**

Total RNA was extracted from cells using RNeasy micro kit (Qiagen, Hilden, Germany) and cDNA was synthesized using the iScriptTM c-DNA Synthesis Kit (Bio-Rad Laboratories Inc., Hercules, CA, USA) according to the manufacturer’s instructions. RT-qPCR was performed using GoTaq qPCR Master Mix (A600A; Promega, Madison, WI, USA). Relative amounts, obtained with 2(-ΔCt) method, were normalized with respect to the housekeeping gene L32. The primer sequences are reported in supplementary materials and methods.

1. **Primer sequences used in this study**

| **Targets** | **Sequence (5’-3’)** |
| --- | --- |
| hsa-miR-155-5p | TTAATGCTAATCGTGATAGGGGTT |
| hsa-miR-342-3p | TCTCACACAGAAATCGCACCCGT |
| hsa-miR-20b-5p | CAAAGTGCTCATAGTGCAGGTAG |
| hsa-miR-17-5p | CAAAGTGCTTACAGTGCAGGTAG |
| hsa-miR-23a-3p | ATCACATTGCCAGGGATTTCC |
| hsa-miR-16-5p | TAGCAGCACGTAAATATTGGCG |
| hsa-miR-106a-5p | AAAAGTGCTTACAGTGCAGGTAG |
| hsa-miR-143-3p | TGAGATGAAGCACTGTAGCTC |
| hsa-miR-9-5p | TCTTTGGTTATCTAGCTGTATGA |
| L32 | F: GGAGCGACTGCTACGGAAG |
| L32 | R: GATACTGTCCAAAAGGCTGGAA |
| SOCS1 | F: AGCGTGAAGATGGCCTCGG |
| SOCS1 | R: GCCGCCACGTAGTGCTCC |
| SOCS5 | F: TCTAAAGCCCTGCCTGCTATT |
| SOCS5 | R: AGTCTCCTATGCTGATGTTTTTCT |
| TGFBR2 | F: GCTCTGGTGCTCTGGGAAAT |
| TGFBR2 | R: GCCTCTGGGTCGTGGTC |
| PIKFYVE | F: GTCCTTCTCATCTCACACACTT |
| PIKFYVE | R: GCCTCCTTCTGCTCTCTCTTTG |
| LDLR | F: GTATCTGTGCCTCCCTGCC |
| LDLR | R: TGGTTGTGTGCTGTGTCCTT |
